# Clinical significance of genetic variation in hypertrophic cardiomyopathy: comparison of computational tools to prioritize missense variants

**DOI:** 10.3389/fcvm.2022.975478

**Published:** 2022-08-18

**Authors:** Pedro Barbosa, Marta Ribeiro, Maria Carmo-Fonseca, Alcides Fonseca

**Affiliations:** ^1^LASIGE, Faculdade de Ciências da Universidade de Lisboa, Lisboa, Portugal; ^2^Instituto de Medicina Molecular João Lobo Antunes, Faculdade de Medicina da Universidade de Lisboa, Lisboa, Portugal; ^3^Department of Bioengineering and iBB-Institute for Bioengineering and Biosciences, Instituto Superior Técnico, Universidade de Lisboa, Lisboa, Portugal; ^4^GenoMed - Diagnósticos de Medicina Molecular, Lisboa, Portugal

**Keywords:** hypertrophic cardiomyopathy, computational pathogenicity prediction, missense variant interpretation, genetic testing, variants-of-unknown-significance, prediction tool comparison

## Abstract

Hypertrophic cardiomyopathy (HCM) is a common heart disease associated with sudden cardiac death. Early diagnosis is critical to identify patients who may benefit from implantable cardioverter defibrillator therapy. Although genetic testing is an integral part of the clinical evaluation and management of patients with HCM and their families, in many cases the genetic analysis fails to identify a disease-causing mutation. This is in part due to difficulties in classifying newly detected rare genetic variants as well as variants-of-unknown-significance (VUS). Multiple computational algorithms have been developed to predict the potential pathogenicity of genetic variants, but their relative performance in HCM has not been comprehensively assessed. Here, we compared the performance of 39 currently available prediction tools in distinguishing between high-confidence HCM-causing missense variants and benign variants, and we developed an easy-to-use-tool to perform variant prediction benchmarks based on annotated VCF files (VETA). Our results show that tool performance increases after HCM-specific calibration of thresholds. After excluding potential biases due to circularity type I issues, we identified ClinPred, MISTIC, FATHMM, MPC and MetaLR as the five best performer tools in discriminating HCM-associated variants. We propose combining these tools in order to prioritize unknown HCM missense variants that should be closely followed-up in the clinic.

## Introduction

Familial hypertrophic cardiomyopathy (HCM) is the most common inherited heart disease and one of the leading causes of sudden cardiac death in younger people (1) and athletes (2). The estimated prevalence of HCM is at least 1 in 500 individuals in the general population (3). Access to more sensitive imaging methods and advanced genetic testing improved the diagnostic rate and a more recent study revealed that 1 in 200 people may be affected (4). In some cases, sudden cardiac death is the first manifestation of HCM, particularly in younger individuals (5–7). Thus, it is important to implement prevention strategies that involve screening, monitoring and counseling HCM patients and their families (8).

HCM is morphologically characterized by increased left ventricular wall thickness in the absence of abnormal loading conditions (9). In the majority of familial HCM patients, the disease is caused by mutations in any of the following eight sarcomeric genes, *MYBPC3, MYH7, TNNT2, TPM1, MYL2, MYL3, TNNI3*, and *ACTC1* (10). Although advances in high-throughput sequencing led to an exponential increase in the number of genes proposed to be associated with HCM, in many cases there is no robust evidence supporting a causative link between these additional genes and the disease (10). Nevertheless, screening of extended gene panels is recommended, including genes associated with other disorders such as inherited metabolic and neuromuscular diseases that may mimic the clinical features of HCM (11).

Genetic testing has become an integral part of the clinical evaluation and management of patients with HCM (12, 13). Detection of a mutation known to be causative of the disease in the index patient is followed by family genetic cascade testing in order to identify which family members do or do not carry the mutation. This allows to eliminate disease risk in non-carrier individuals, and to implement primary prevention strategies in individuals with pre-symptomatic genetic diagnosis (9). With contemporary disease management, approximately two thirds of patients with HCM have a normal life expectancy without significant morbidity, while a subset requires symptomatic therapies for heart failure (14).

In some patients, however, no causative mutation is identified. A recent systematic review and meta-analysis revealed a mutation detection rate of 33–43% in adult HCM cohorts and 52–78% in pediatric HCM cohorts (15). The detection rates for adult cohorts with a positive family history of HCM were significantly higher compared with apparently sporadic cases, whereas in pediatric cohorts the detection rate was similar irrespective of family history (15). Approximately 40% of HCM patients were reported as presenting a non-familial subtype for which the underlying mechanism remains unknown (16, 17).

In addition to ambiguous gene associations, difficulties in classifying variants in “core” HCM genes limits the impact of genetic testing in clinical practice (4). Determining which genetic variants detected in HCM-associated genes are pathogenic relies on a set of functional (molecular) and clinical criteria that have been defined by the American College of Medical Genetics and Genomics and the Association for Molecular Pathology (ACMG/AMP) (18–20). In many cases, available information is insufficient to classify a variant as benign/likely benign or pathogenic/likely pathogenic. This results in a large group of so-called “variants-of-unknown-significance” (VUS), the interpretation of which is extremely challenging. The advent of whole-exome and whole-genome databases revealed that many variants previously associated with cardiomyopathies were rather likely benign, as their population frequencies were incompatible with the prevalence of disease (21, 22). These observations prompted the development of disease-specific approaches to assist decisions on which variants should be considered in clinical practice. Rigorous curation efforts that assess all available lines of evidence for HCM-association are now available, such as the SHaRe registry (https://theshareregistry.org/), which comprises genetic data and cardiac morphofunctional parameters for >9,000 HCM patients.

When a VUS or a previously unseen genetic variant is identified in an HCM-causing gene, computational approaches can aid by making a prediction of potential pathogenicity. Over the last decade, many different algorithms and tools have been proposed, but their performance is not consistent across different independent benchmarks (23–31). Additionally, most of these studies evaluate prediction tools on datasets that incorporate variants from multiple disease phenotypes, which may compromise their performance for a specific disease (27, 32, 33).

In this study, we used three distinct datasets comprising high-confidence HCM-causing missense variants and we developed a dedicated computational framework (VETA) to perform a comprehensive analysis of currently available prediction algorithms. We found that ClinPred, MISTIC, FATHMM, MPC and MetaLR are the five tools that more accurately and reliably distinguish between benign and HCM-causing missense variants.

## Methods

### Prediction scores

When available, pre-computed prediction scores were obtained directly from each tool website. Alternatively, dbNSFP v4.0b1 (34) was used. UCSC genome browser was used to access conservation scores (35).Because some tools do not provide scores for the latest genome build, the GRCh37 version was used to include a more comprehensive number of tools in the analysis. Annotation of VCF scores was performed with Ensembl Variant Effect Predictor (VEP) v105 (36) using custom plugins or with vcfanno v0.3.3 (37). Reference tool thresholds were obtained from different sources according to the following priority. First, we followed a recent computational approach that calibrates missense variants' thresholds to different levels of pathogenicity evidence according to the ACMG/AMP guidelines (33). Whenever reported in this dataset, we used the least conservative value in the threshold range with “Supporting” evidence for Pathogenicity (PP3). Alternatively, we used the threshold value provided in the original publication, or indicated by authors elsewhere (e.g., online repository for the tool data). If not available, we included threshold values indicated in other studies that use the tool (e.g., benchmark paper).

### Datasets

#### ClinVar HCM

The ClinVar v20220403 database (38) was used. To select HCM-associated variants, we filtered the dataset using a combination of three disease ontologies by keeping variants with any of the following identifiers: MedGen (39) (C3495498, C0949658); OMIM (40) (192600); Mondo Disease Ontology (41) (0005045, 0024573). Additionally, we excluded all variants with zero-star review status, classified as “Uncertain_significance” or with conflicting interpretations of pathogenicity. Finally, missense variants were selected by inspecting the “Consequence” field of VEP annotations. The resulting HCM ClinVar dataset consisted of 471 missense variants (278 Pathogenic/Likely_pathoenic, 193 Benign/Likely_benign; Supplementary Table S1).

**Figure 1:**
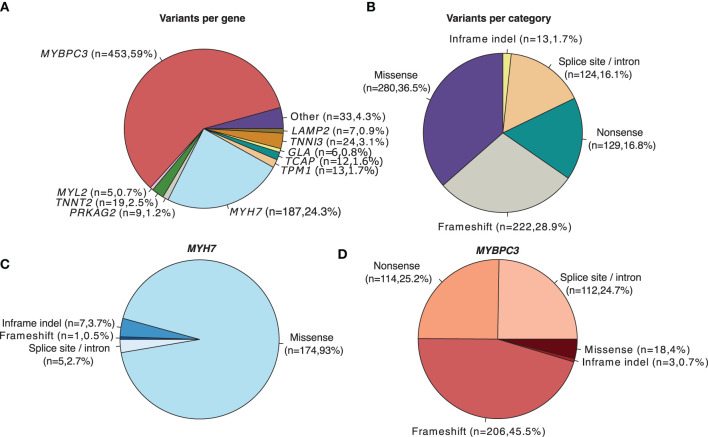
Distribution of HCM-associated variants (Pathogenic/Likely pathogenic) with a review status of > 1 star in ClinVar (*N* = 768). **(A)** Number and proportion of overall variants per gene. **(B)** Number and proportion of overall variants per category. **(C)** Category of variants located in the *MYH7* gene. **(D)** Category of variants located in the *MYBPC3* gene.

For the variant distribution analysis depicted in Figure 1, the VCF field GENEINFO was used to assign the gene name. Genes with <5 variants were grouped as “Other”. Variant categories were extracted from the VCF MC field. For the cases where MC was empty, Consequence field from VEP annotations was used instead. “inframe_deletion” and “inframe_insertion” ontologies were generalized to “Inframe indel”. Splice site and “intron_variant” annotations were combined into one single category “Splice site/Intron”.

#### SHaRE cohort

Data was directly accessed from (17). Variants with Pathogenic/Likely pathogenic assignments that were absent from the ClinVar dataset were selected. Records with allele mismatch after running Ensembl VEP were excluded. The majority of remaining variants refer to either missense or splice region variants. Missense variants were selected as described above. After this selection, 93 HCM-associated missense variants were considered for further analysis (Supplementary Table S2).

#### Walsh_2017

This dataset was generated from results in (22). In this study, the following genes showed a significant excess of rare variation in patients compared to control individuals: *ACTC1, FHL1, GLA, MYBPC, MYH7, MYL2, MYL3, PRKAG2, TNNI3, TNNT2, TPM1*. We selected the variants considered Pathogenic/Likely_pathogenic, excluding those with conflicting interpretations between different labs. A final set of 103 missense variants was obtained (Supplementary Table S3).

#### gnomAD

gnomAD v2.1 (42) was used to identify benign variants for a balanced evaluation of the SHaRe and Walsh_2017 datasets. Common variants were selected based on a Minimum Allele Frequency (MAF) threshold of 0.001 (>0.1%). We restricted our analysis to variants located in 62 genes associated with cardiomyopathies (https://www.ncbi.nlm.nih.gov/gtr/tests/509149/). From a total of 110,762 variants, 709 missense variants were further selected. Hits in the *TTN* gene were discarded to avoid over-representation of missense variants from a single gene. Finally, we checked that the remaining 356 variants did not overlap with any variant from the other datasets, as well as with variants from the whole ClinVar database with any of the following assignments: “Pathogenic”, “Likely pathogenic”, “Pathogenic/Likely Pathogenic”, “Uncertain significance”, or “Conflicting interpretations of pathogenicity”. The final set comprised 220 variants, which were randomly split in two non-overlapping groups of 100 variants, called benign_set1 (used to compare with the SHaRe HCM dataset; Supplementary Table S2) and benign_set2 (used to compare with the Walsh_2017 HCM dataset; Supplementary Table S3).

### Performance metrics

For each tool, a confusion matrix was constructed that measures the number of True Positives (TP), True Negatives (TN), False Positives (FP) and False Negatives (FN). TP refers to the number of pathogenic variants that a tool correctly predicts to be pathogenic (e.g. above the reference threshold). TN is the number of benign variants that a tool correctly predicts to be benign; FP indicates the number of benign variants that a tool predicts to be pathogenic; FN is the number of pathogenic variants that a tool predicts to be benign. Tools were ranked using a small variation of the Matthews correlation coefficient ($MCC\;=\;TP^{*}TN-FP^{*}FN\;/\;\sqrt{\left(TP+FP)(TP+FN)(TN+FP)(TN+FN\right)}$). To account for the magnitude of missing predictions, MCC values were normalized to range from 0 and 1 ($normalizedMCC\;=\;\frac{\left(MCC+1\right)}{2}$), and weighted by the fraction of variants that a tool gives predictions (tool coverage). Throughout the manuscript we call this metric weighted normalized MCC, which corresponds to **weighted norm** **MCC** **=** coverage^*^normalizedMCC. In addition, tools were ranked based on Receiving Operating Characteristic (ROC) curves, which, as opposed to weighted normalized MCC, evaluate performance at multiple threshold values. ROC curves were created by plotting the Sensitivity (also known as True Positive Rate or Recall) against the 1—Specificity (also known as False Positive Rate) at several different thresholds. **Sensitivity=TP/TP+FN**; **Specificity=TN/TN+FP**. For each tool, scores were transformed based on their rank so that they ranged between 0 and 1 (in the minority of tools where the values below a threshold are considered pathogenic, we inverted the signal accordingly). The area under the ROC curve (auROC) was used as the summary statistic.

#### Automated analysis using VETA

Most of the analysis in the manuscript were performed with Variant prEdiction Tools evAluation (VETA), a general tool we developed to benchmark variant predictors. Briefly, VETA takes annotated VCF files from Ensembl VEP (36) as input (in this analysis we specifically set –hgvs, –per_gene, –pick_order ccds, canonical, biotype, rank, –no_intergenic –gencode_basic) and automatically compares tools performance at different levels. It allows to evaluate predictions according to the variant type (e.g., SNVs, indels), variant location (e.g., exons, introns) and scope of the tool (e.g., separate analysis for missense and splicing predictors). In addition, VETA is particularly suited to deal with ClinVar data since it incorporates methods to filter variants according to review status and/or phenotype desired. Furthermore, VETA is able to inspect whether reference thresholds are appropriate, and allows combination of scores from multiple tools to create meta-predictors using standard Machine Learning algorithms. By default, VETA has native support for more than 50 predictors, but it also allows users to include custom tools through a configuration file. Detailed documentation is available at https://github.com/PedroBarbosa/VETA, where instructions for easy installation are provided. Of note, VETA depends on cyvcf2 (43) for VCF parsing, on hgvs (44) for parsing HGVS expressions, on Scikit-learn for ROC curve analysis (45) and seaborn for plots generation (46). Importantly, VETA does not run any prediction tool but rather receives as input VCF files annotated with prediction scores.

#### Threshold analysis

To evaluate whether published reference thresholds were appropriate, we measured the performance of each tool using a set of 100 threshold values uniformly distributed over the observed range of scores. The best thresholds were obtained based on the F-Beta formula $F_{\beta }\;=\;\left(1+\;\beta^{2}\right)\;\frac{Precision\;\times \;Recall}{\left(\beta^{2}+Precision\right)+Recall}$, where **Precision=TP/TP+FP**. This is similar to the commonly used F1 score, but allows to weight the balance between precision and recall using the β parameter (when β = 1, it is equal to the F1 score). In this study, we used β = 0.5, 1 and 1.5. For each β value, the threshold that maximized the Fβ function was selected. Higher β values favor sensitivity over precision, which translate into higher recall rates at the cost of increasing false positives. Conversely, lower β values favor precision, at the cost of increasing false negatives.

We additionally performed a bootstrapping procedure to evaluate how reliable the adjusted threshold is. For each tool, we generated 1,000 bootstrap samples of the same size of the data sample with the same ratio of pathogenic/benign variants as in the original dataset. For each bootstrap sample we derived the best threshold (as described above). Then, we computed the 0.025 and 0.975 quantiles of the distribution of the bootstrap sample statistic (distribution of best thresholds). We used these values to interrogate at which threshold range 95% of the bootstrap sample statistic lies, and how wide/narrow this interval is in respect to the adjusted threshold originally obtained.

## Results

### The majority of HCM-associated variants annotated in ClinVar are missense

Among algorithms developed to assess the likelihood of pathogenicity of rare variants, two main categories are generally considered: those that predict whether a missense change (i.e., a base change that alters the encoded amino acid) is damaging to the resultant protein function or structure and those that predict whether there is an effect on splicing (18). To determine the relative contribution of missense variation to HCM, we analyzed all HCM-associated variants annotated in the ClinVar database that are classified as Pathogenic/Likely pathogenic with a review status of > 1 star (Figure 1). As previously described (47), the most frequently mutated genes are *MYBPC3* and *MYH7* (Figure 1A). Among all HCM-associated variants, missense variants are the most frequent (Figure 1B). However, the prevalence of missense variants differs depending on the affected gene. Over 90% of annotated variants in the *MYH7* gene are missense (Figure 1C), whereas in the *MYBPC3* gene missense variants are less than 20% (Figure 1D).

### Comprehensive review of computational tools to predict clinical significance of missense variants

Having shown that missense variants are a frequent cause of HCM, we next performed a comprehensive review of computational tools that predict the clinical significance of this type of genetic change (Table 1). A subset of existing computational tools relies on features such as amino acid or nucleotide conservation, the location and context within the protein sequence, and the biochemical consequence of the amino acid substitution (see references in Table 1, “protein predictors”). Other methods estimate the probability that a particular nucleotide belongs to a conserved element irrespective of its location in the genome, and therefore are not restricted to variation in exons of protein coding genes (see references in Table 1, “conservation scores”). Another class consists of tools that integrate genome-wide features to predict variant effects irrespective of the variant category (Table 1, “Consequence-agnostic predictors”). Tools specifically designed for cardiac diseases were also included (Table 1, “disease-specific predictors”).

**Table 1:** Prediction tools analyzed in this study.

**Category**	**Tool**	**Threshold**
Protein predictors	SIFT (48)	<0.01 (33)
	MutPred (49)	>0.5*
	PolyPhen-2 HDIV (50)	>0.978 (33)
	PolyPhen-2 HVAR (50)	>0.978 (33)
	Mutation Assessor (51)	>1.935 (52)
	Condel (53)	>0.98 (53)
	VEST4 (54)	>0.764 (33)
	MutationTaster2 (55)	>0.5 (52)
	FATHMM (56)	<-4.14 (33)
	PROVEAN (57)	<-2.5 (52)
	MetaSVM (25)	>0.5 (25)
	MetaLR (25)	>0.5 (25)
	M-CAP (58)	>0.025 (58)
	REVEL (59)	>0.644 (33)
	MPC (60)	>1.360 (33)
	MTR (61)	<0.5*
	PrimateAI (62)	>0.790 (33)
	ClinPred (63)	>0.5 (63)
	MISTIC (64)	>0.5 (63)
	cVEP (65)	>0.5**
	MVP (66)	>0.7 (63)
	VARITY (67)	>0.75 (67)
	MutFormer (68)	>0.5*
	EVE (69)	>0.5***
	MutScore (70)	>0.5*
Conservation scores	phastCons (71)	>0.99 (28)
	phyloP (72)	>7.367 (33)
	SiPhy (73)	>12.7 (25)
	GERP (74)	>4.4 (25)
	CDTS (75)	<10 (75)
Consequence-agnostic predictors	GWAVA (76)	>0.4 (77)
	FATHMM-MKL (78)	>0.5 (52)
	DANN (79)	>0.9 (80)
	Eigen (81)	>1 (58)
	ReMM (82)	>0.984 (83)
	CAPICE (84)	>0.02 (84)
	CADD (85)	>25.3 (33)
Disease-specific predictors	CardioVAI (86)	>2 (86)
	CardioBoost (32)	>0.9 (32)

* If a reference threshold was not found, decision boundary was set to 0.5 for tools with a score range between 0 and 1.

** cVEP outputs categorical labels (e.g. Pathogenic, Likely_benign). We transformed categories into numerical predictions to allow doing the benchmark as following: Benign: 0; Likely_benign: 0.25; Likely_pathogenic: 0.75; Pathogenic: 1. VUS classifications were treated as NaN. Since these transformations represent artificial numeric predictions, this tool was just used in the first comparison, where tools are evaluated according to reference cut-offs. Downstream analysis (e.g. best threshold analysis, ROC curves) did not include cVEP.

*** For EVE, we tested doing the benchmarks using the categorical classifications at three different uncertainty thresholds (20, 82, 87). We transformed categorical classifications as we did for cVEP. At the end, we observed that none of these annotations improved classifications compared with using the raw EVE numeric score. For initial performance assessment, we set EVE threshold to 0.5, as defined in^**^.

### Threshold optimization for HCM-associated variants

**Figure 2:**
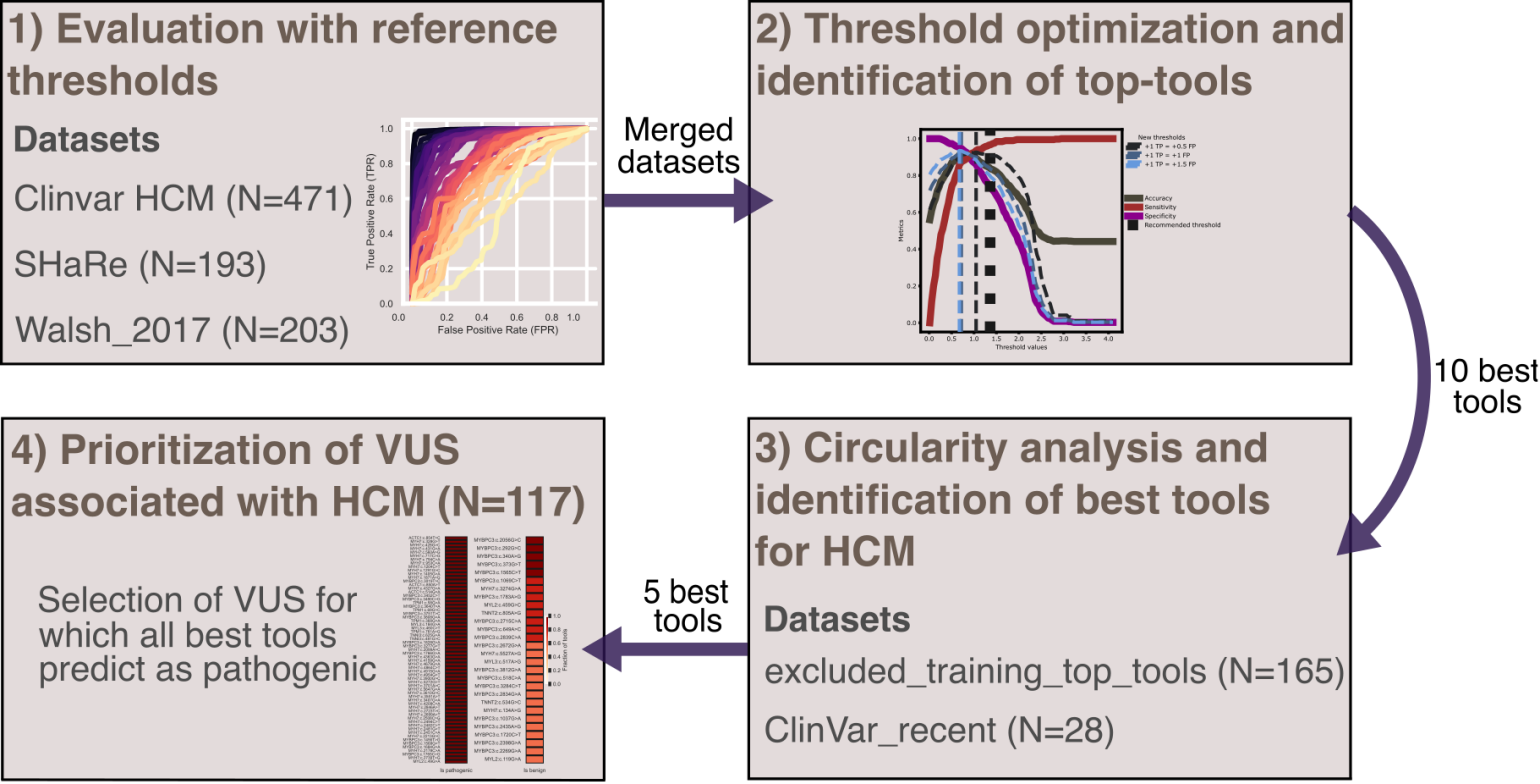
Workflow of the study. Number of variants on each dataset are presented.

**Figure 3:**
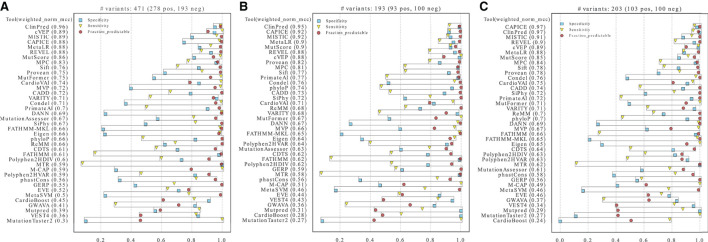
Performance of prediction tools in classifying HCM missense variants using fixed thresholds for ClinVar **(A)**, SHaRe **(B)** and Walsh_2017 **(C)** datasets. For each dataset, the numbers of pathogenic/likely pathogenic (N pos) and benign/likely benign (N neg) variants are indicated. Tools were ranked according to the weighted normalized MCC (weighted_norm_mcc).

To compare the performance of the different tools in classifying pathogenic and benign missense variants, we used three distinct high-confidence HCM test datasets that are mostly based on expert-reviewed clinical and functional evidence (Figure 2). We first assessed performance on the three datasets using the threshold recommended by each tool (Figure 3). The results show that ClinPred, CAPICE, cVEP, MISTIC, MetaLR, REVEL and MutScore consistently ranked among the best tools with weighted normalized MCC values >0.80 (Figures 3A–C). These tools scored with high sensitivity and specificity (approximately 80% or higher) and provided predictions for the vast majority (>90%) of the variants analyzed. Other tools, such as VEST4, ranked worse despite scoring with relatively high sensitivity and specificity because they failed to provide predictions for many of the variants analyzed. A subset of tools, including cardiac-specific methods, failed to predict predominantly benign variants (Supplementary Figure S1).

**Figure 4:**
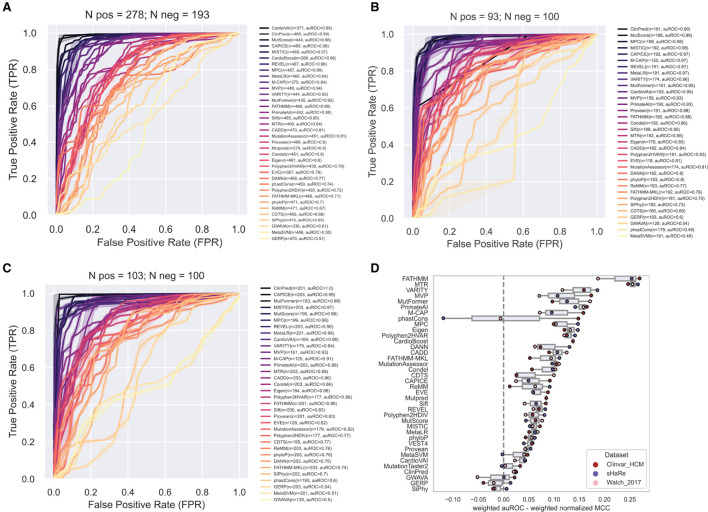
Performance of prediction tools in classifying HCM missense variants using ROC curve analysis for ClinVar **(A)**, SHaRe **(B)** and Walsh_2017 **(C)** datasets. For each dataset, the numbers of pathogenic/likely pathogenic (N pos) and benign/likely benign (N neg) variants are indicated. Tools were ranked according to the area under the ROC curve (auROC). The number (n) of variants predicted by each tool is indicated. Tools with more than 50% of missing predictions were not included. **(D)** Differences in the metrics when evaluating with auROC and weighted normalized MCC. For comparison, auROC values were weighted by the fraction of variants predicted by each tool.

In contrast, ROC curves (calculated only from scored variants) revealed overall excellent performance, with several tools with auROC scores above 0.9 (Figures 4A–C). This analysis highlights how much results can change depending on the selected metric. For example, CardioVAI, which was ranked in an intermediate position using MCC-based values for the recommended thresholds (see Figure 3), appears as one of the best in the ROC curve analysis (auROC ≥ 0.95). An overall comparison of auROC (from multi-threshold analysis) and MCC (from fixed threshold analysis) scores reveals performance differences within each tool clearly favoring auROC, which indicates that reference thresholds of several methods may be suboptimal for HCM (Figure 4D).

**Figure 5:**
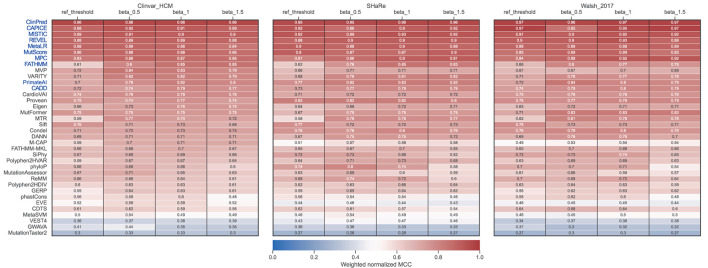
Performance of prediction tools using adjusted thresholds on each dataset (ClinVar, SHaRe, and Walsh_2017). Optimized thresholds at Beta = 0.5 minimize the false positives (variants predicted as pathogenic that are benign). Optimized thresholds at Beta = 1 give the same importance to false positives and false negatives. Optimized thresholds at Beta = 1.5 minimize the false negatives (variants predicted as benign that are pathogenic). Tools highlighted in blue were selected as the best by averaging the ranks between the three datasets.

This observation prompted us to carry out a threshold analysis to find values that best discriminate the high-quality pathogenic HCM variants. We merged the three datasets, leading to 867 variants (474 pathogenic, 393 benign). Using the F-Beta (β) score at different values of β, we derived new thresholds that prioritize differently precision and recall (Table 2). Next, we evaluated tools performance using the adjusted thresholds on each dataset independently (Figure 5). The results confirm improved performance. Yet, no major change was observed for the top ranked tools. Notably, the performance of top tools remained similar when different weights to precision/recall were given. Although different types of errors were introduced using different thresholds, these results suggest that the top tools are similarly sensitive to type I (more false positives at β of 1.5) and type II errors (more false negatives at β of 0.5).

**Table 2:** Adjusted thresholds that maximize performance for HCM variants at different levels of importance given to precision and recall.

Tool*	**Reference threshold**	Threshold_beta_0.5**	Threshold_beta_1**	Threshold_beta_1.5**
**ClinPred**	0.5	0.52 (0.366, 0.832)	0.41 (0.242, 0.533)	**0.37 (0.123, 0.476)**
**CAPICE**	0.02	**0.61 (0.116, 0.619)**	0.06 (0.016, 0.078)	0.02 (0.009, 0.058)
**MISTIC**	0.5	**0.67 (0.6, 0.781)**	0.543 (0.499, 0.611)	0.514 (0.395, 0.544)
**REVEL**	0.644	0.596 (0.533, 0.679)	**0.441 (0.407, 0.565)**	**0.353 (0.323, 0.473)**
**MPC**	1.36	**1.047 (0.808, 1.281)**	**0.717 (0.672, 0.878)**	**0.675 (0.572, 0.774)**
**MetaLR**	0.5	0.629 (0.547, 0.658)	0.509 (0.346, 0.606)	**0.26 (0.212, 0.491)**
**MutScore**	0.5	**0.75 (0.714, 0.818)**	**0.741 (0.512, 0.757)**	0.501 (0.38, 0.582)
**FATHMM**	−4.14	–**2.119 (**–**2.156**, –**1.062)**	–**1.078 (**–**1.185**, –**0.759)**	–**0.947 (**–**1.137**, –**0.209)**
**PrimateAI**	0.79	**0.693 (0.641, 0.733)**	**0.577 (0.552, 0.645)**	**0.524 (0.504, 0.583)**
**CADD**	25.3	**24.12 (22.915, 24.742)**	**23.04 (22.055, 23.348)**	**22.32 (21.336, 23.066)**
**VARITY**	0.75	**0.427 (0.375, 0.597)**	**0.348 (0.243, 0.428)**	**0.23 (0.169, 0.299)**
**Provean**	−2.5	−2.582 (−2.932, −2.182)	–**2.268 (**–**2.423**, –**1.507)**	–**1.484 (**–**2.215**, –**0.946)**
**MutFormer**	0.5	**0.99 (0.96, 0.998)**	**0.98 (0.954, 0.993)**	**0.98 (0.664, 0.988)**
**Condel**	0.468	**0.79 (0.624, 0.866)**	**0.59 (0.484, 0.671)**	0.47 (0.463, 0.561)
**MTR**	0.5	**0.783 (0.746, 0.817)**	**0.883 (0.815, 0.917)**	**0.916 (0.883, 0.931)**
**CardioVAI**	2	**2.53 (2.502, 2.852)**	**2.53 (2.502, 2.852)**	2.53 (1.515, 2.837)
**DANN**	0.9	**0.991 (0.964, 0.997)**	**0.991 (0.956, 0.994)**	**0.959 (0.903, 0.99)**
**MVP**	0.7	**0.891 (0.873, 0.924)**	**0.862 (0.818, 0.893)**	**0.793 (0.778, 0.861)**
**Sift**	0.001	0.0 (0.001, 0.038)	**0.05 (0.003, 0.096)**	**0.13 (0.043, 0.196)**
**Eigen**	1	**4.282 (2.973, 5.641)**	**2.531 (2.275, 3.155)**	**2.337 (1.384, 2.777)**
**SiPhy**	12.17	11.823 (10.859, 13.018)	**10.398 (9.507, 11.931)**	**7.264 (6.245, 10.729)**
**phyloP**	7.367	**7.012 (4.39, 7.122)**	**3.558 (0.76, 4.512)**	**0.105 (0.022, 1.068)**
**Polyphen2HVAR**	0.978	**0.65 (0.359, 0.861)**	**0.24 (0.156, 0.463)**	**0.02 (0.012, 0.265)**
FATHMM-MKL	0.5	**0.978 (0.948, 0.986)**	**0.959 (0.58, 0.964)**	0.464 (0.436, 0.881)
ReMM	0.984	0.98 (0.943, 0.989)	**0.88 (0.84, 0.95)**	**0.341 (0.313, 0.886)**
Polyphen2HDIV	0.978	**0.94 (0.511, 0.957)**	**0.5 (0.021, 0.565)**	**0.02 (0.0, 0.064)**
GERP	4.4	**3.401 (2.602, 3.601)**	**2.583 (2.203, 3.388)**	**2.232 (0.057, 2.679)**
MutationAssessor	1.935	**2.462 (2.429, 2.828)**	1.106 (0.915, 2.258)	**0.905 (0.085, 1.122)**
M-CAP	0.025	**0.181 (0.127, 0.332)**	**0.131 (0.074, 0.162)**	**0.131 (0.051, 0.142)**
CDTS	10	**26.756 (6.601, 37.161)**	**62.386 (37.155, 85.472)**	**86.139 (62.899, 94.343)**
phastCons	0.99	0.7 (0.532, 1.0)	**0.54 (0.256, 0.809)**	**0.001 (0.001, 0.692)**
MetaSVM	0.5	**0.106 (0.015, 0.363)**	**0.011 (0.012, 0.135)**	**0.011 (0.012, 0.109)**
EVE	0.5	0.291 (0.264, 0.515)	**0.252 (0.146, 0.295)**	**0.127 (0.062, 0.2)**
VEST4	0.764	**0.662 (0.484, 0.733)**	**0.504 (0.385, 0.59)**	**0.445 (0.369, 0.522)**
GWAVA	0.5	**0.294 (0.239, 0.475)**	**0.07 (0.071, 0.296)**	**0.07 (0.07, 0.215)**
MutationTaster2	0.5	**0.99 (0.742, 0.999)**	0.99 (0.228, 0.992)	0.23 (0.033, 0.987)

95% percentile values of the bootstrap distribution are also displayed. Mutpred and CardioBoost were not included since they did not predict the minimum number of variants (N = 50) in the minority class required by VETA for threshold analysis.

* Tool names in bold represent those that display minimally useful predictive power (> 0.70 weighted normalized MCC) across the different datasets (Figure 5).

** Numbers in bold represent cases for which the reference threshold lies outside the 95% percentile values of the bootstrap distribution of adjusted thresholds.

Contrasting with the top tools, an improvement of overall predictions was observed for the middle-ranked tools after threshold optimization (Figure 5). For example, VARITY, FATHMM and MTR had a >10% increase of the weighted normalized MCC values for almost all the dataset/threshold combinations. For the lowest performing tools, threshold optimization has no significant effect (e.g. GWAVA, MetaSVM, GERP; Figures 4, 5). In addition, tools with a large fraction of missing predictions rank poorly, regardless of the threshold optimization (e.g. Mutpred, VEST4, CardioBoost; Figures 3, 5).

### Addressing circularity

Circularity is a critical issue to be considered when assessing performance metrics (88). In this regard, we interrogated whether some of the variants present in our evaluation datasets had been previously used for tool training (Type I circularity). We restricted circularity analysis to the best performing tools. The rank of each tool across all datasets was averaged, and the 10 tools with the lower average rank value with adjusted thresholds at β = 1 were selected. These included ClinPred, MISTIC, CAPICE, REVEL, MetaLR, MPC, MutScore, PrimateAI, FATHMM and CADD (Figure 5).

**Figure 6:**
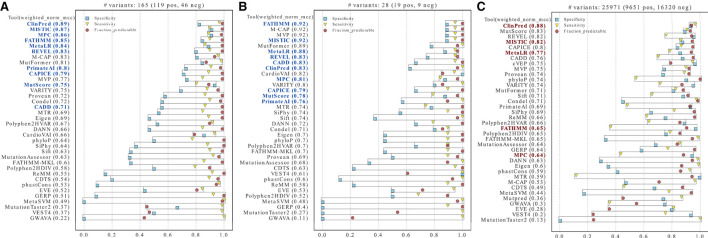
Performance of prediction tools after addressing circularity issues. Tools were ranked using the weighted normalized MCC on two new test datasets **(A–C)**. **(A)** Variants identified as present in the training sets of the tools highlighted in blue were removed from the merged ClinVar, SHaRe, and Walsh_2017 datasets. **(B)** HCM ClinVar variants submitted after the tools highlighted in blue were developed. **(C)** Variants in the whole ClinVar irrespective of disease context. The tools selected as best-performers for HCM are highlighted in red (bold).

For some tools, circularity could not be properly addressed because the training datasets were not explicitly available. This was the case for the pathogenic sets of REVEL, FATHMM and MISTIC (in this case, partially), which used HGMD (89) variants undisclosed for licensing reasons. For other unavailable sets (such as the benign variants of FATHHM and MPC, and both benign and pathogenic datasets of ClinPred), we tried to replicate the data generation process following the methods of each publication, but we cannot ensure that the original sets were accurately reproduced. All VCF files generated (except for CADD that was downloaded directly from the website) are available at https://github.com/PedroBarbosa/paper_HCM_benchmark. Finally, we generated a new test dataset termed “excluded_training_top_tools set”, where variants present in the training of top-performant tools were excluded (Supplementary Table S4). As expected, performance decreased after correcting for type I circularity, with no tool achieving a weighted normalized MCC of 0.9 (Figure 6A). Nevertheless, four tools display metric values above 0.85 (ClinPred, MISTIC, MPC, FATHMM). Notably, these tools still perform better than most of the others, including those that were not controlled for circularity (Figure 6A).

As an alternative approach to address circularity, we analyzed specifically ClinVar variants that were reported after the tools under assessment were developed (“ClinVar_recent set”, Supplementary Table S5). Despite the very low number of variants and the partial overlap with the “excluded_training_top_tools” set, this dataset has the advantage of better controlling for bias favoring any tool for which no training data is available. The results show weighted normalized MCC values above 0.90 for FATHMM and MISTIC, and values above 0.80 for MetaLR, REVEL, CADD, ClinPred, and MPC (Figure 6B). Additional tools with evaluation scores above 0.80 include M-CAP, MVP and MutFormer, which partially use HGMD variants in their training datasets and therefore could not be controlled for potential biases.

Having addressed type I circularity issues, we next identified the five best-performing tools by measuring the average of the tools' ranks in Figures 6A,B, considering each dataset size (i.e., more weight was given to the “excluded_training_top_tools” set). The resulting list includes ClinPred, MISTIC, FATHMM, MPC and MetaLR. We further observed a high correlation between the predictions of these best-performing tools (Supplementary Figure S2).

We next asked how the best tools in discriminating between HCM-associated and benign missense variants perform in classifying missense variants irrespectively of the disease context. We selected missense variants in the whole ClinVar database and we excluded variants that were used in the training of the tools for which we addressed circularity. The resulting dataset consists of 25,971 missense variants (9,651 Pathogenic/Likely_pathogenic, and 16,320 Benign/Likely_benign, Supplementary Table S6). The analysis was performed with the previously recommended thresholds (as indicated in Table 1). Overall, the MCC scores are lower compared to the HCM datasets (pval = 0.036, one-sided Wilcoxon signed-rank test), highlighting the value of disease-specific analysis. Notably, a subset of the best tools selected for HCM (namely, ClinPred and MISTIC) still scored with MCC values > 0.80 (Figure 6C).

### High-confidence prioritization of HCM-associated VUS

**Figure 7:**
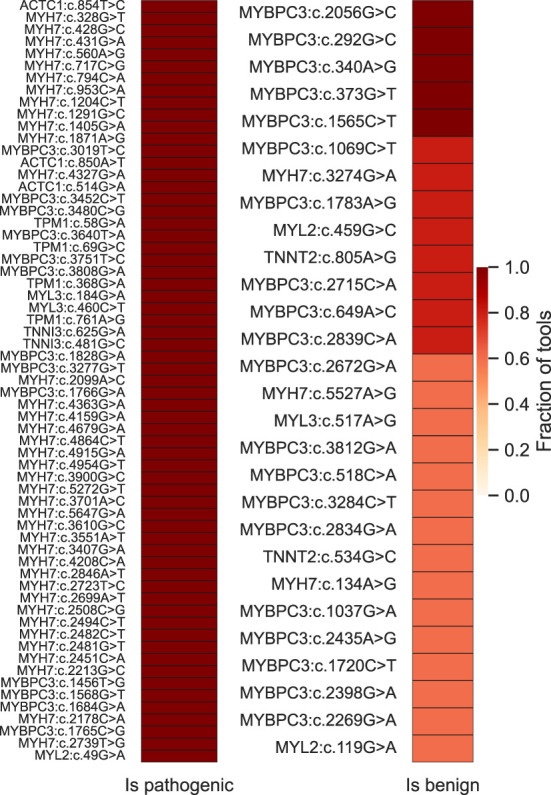
High-confidence prioritization of HCM-associated VUS based on predictions of the 5 top-performant tools (ClinPred, MISTIC, FATHMM, MPC and MetaLR). On the left, 63 variants for which 100% of the tools predict pathogenicity. On the right, variants predicted to be benign by more than 50% of the tools.

Finally, we used the five best-performing tools (ClinPred, MISTIC, FATHMM, MPC and MetaLR) to inspect a non-redundant set of HCM-associated variants classified as VUS in the SHaRe (*N* = 103) and Walsh_2017 (*N* = 14) datasets (Supplementary Table S7). For those variants annotated in ClinVar, we confirmed they remain classified as VUS (as of April 2022). The results show that the majority (81%) of the variants are predicted to be pathogenic by more than 50% of the tools. Particularly, 63 variants were predicted to be pathogenic by all the tools, most of them located in the *MYH7* gene (Figure 7). We additionally inspected predictions on *MYH7* VUS made by CardioVAI, which considers the ClinGen Expert Panel adaptation of ACMG/AMP guidelines for MYH7 variants (87). The results are consistent with the pathogenic predictions of the top 5 tools, except for two variants predicted as benign by CardioVAI (c.3701A>C and c.3551A>T). Given the high-agreement level of classifications, we propose that variants highlighted in Figure 7 should be prioritized for further clinical and functional studies.

## Discussion

The accurate identification of genetic changes associated with increased risk for HCM remains challenging. According to the ACMG/AMP guidelines, computational predictions are included as one line of evidence to assess the clinical significance of genetic variation (18–20). Although many computational tools are currently available, it is unclear which should be selected for clinical genome interpretation. To date, multiple independent variant prediction benchmarking studies have been published (23–31). However, the results are usually not consistent, and one reason for this discrepancy may relate to the different benchmark datasets used (27, 32). In addition, these studies do not evaluate prediction tools on a disease-specific manner [except for (27)]. It is also common that performance is evaluated using the receiver operating characteristic (ROC) curve, which does not reflect the fixed thresholds used in medical genetic testing (32). Moreover, the frequently used dbNSFP resource lacks more recently developed approaches (68, 69).

In this study, we developed a computational framework (VETA) to compare the performance of 39 algorithms in predicting missense variants known to be implicated in the pathogenesis of a specific disease, HCM. We focused on missense variants because HCM is frequently caused by this type of genetic variation (Figure 1B). HCM-associated missense variants may disrupt normal sarcomeric assembly and function by changing an amino acid in a highly-conserved protein residue, altering important kinase domains that affect ligand interaction, or changing surface-exposed residues that affect protein-protein interaction (47). Missense variants can also cause protein misfolding and accelerated degradation, thus leading to haploinsufficiency (47). As “ground-truth” for prediction assessment, we used datasets of HCM-associated missense variants classified with high-confidence based on expert-reviewed clinical and functional evidence.

We included the fraction of missing scores in performance metrics, and this clearly influenced the ranking (Figure 3). In particular, cardiac-specific methods such as CardioVAI and CardioBoost, were designed to predict variants in “core” disease-linked genes such as *MYH7*. Many of the genes included in our benign dataset differ from those used by CardioVAI and CardioBoost and this is probably the reason why these tools failed to score multiple “ground-truth” benign variants in our analysis (Supplementary Figure S1).

Another contribution of our study is the calculation of new thresholds specifically calibrated for HCM. We found all tools with predictive power above 0.7 (regarded as minimally useful) to have a recommended threshold that falls outside of the bootstrap estimate interval of the tuned thresholds (Table 2, bold tools and values). This reveals that previously reported reference thresholds are not ideal for application to HCM-related variants. To enable flexibility in the choice of new thresholds, we derived adjusted values where importance given to recall of precision varies. If the goal is to maximize the identification of pathogenic variants, one must use the threshold obtained using a Beta value of 1.5. However, for missense HCM variants, we observed that most errors come at the cost of lower specificity (benign variant predicted as harmful. Figures 6A,B), thus it might be preferable to use thresholds obtained at a Beta value of 0.5, especially when looking at rare missense variants.

After tackling potential biases related to circularity issues, we identified ClinPred, MISTIC, FATHMM, MPC and MetaLR as the five best performers. ClinPred (63) incorporates two machine learning algorithms that use existing conservation, pathogenicity scores and population allele frequency from the gnomAD database as input features. MISTIC (64) combines two complementary machine learning algorithms using a soft voting system that integrates 113 missense features, ranging from allele frequencies from the Exome Aggregation Consortium (ExAC) and conservation/pathogenicity scores, to physiochemical and biochemical properties of amino acids. FATHMM (56) builds Hidden Markov models from multiple sequence alignments along with pathogenicity weights to predict the functional, molecular, and phenotypic consequences of amino acid substitutions. MPC (60) is a deleteriousness metric that incorporates depletion of missense variation across genes by leveraging the sequencing data from ExAC (60,706 individuals). Finally, MetaLR (25) is a Logistic Regression model that integrates multiple scoring methods. Thus, most of these top-ranked tools integrate several previous models as features in their algorithms, and their predictions tend to be highly correlated (Supplementary Figure S2). These results are in line with previous observations indicating that meta-predictors tend to perform better than individual counterparts (31, 90).

While some experts argue that VUS reporting may lead to confusion and cause more harm than benefit to the patient and family (91), others highlight the importance of appropriate clinical follow-up as it may contribute to clarify the variant's impact and eventually lead to its reclassification (92). We propose combining the best performing tools identified in this study to provide clinicians with a high-confidence prioritization of VUS and newly detected variants identified by genetic testing in HCM patients. Testing family members for a prioritized variant may reveal its presence in multiple affected individuals and absence in healthy individuals, indicating that the variant should be considered pathogenic.

Several lines of evidence indicate that a disease-specific approach improves variant interpretation, namely in inherited cardiac disorders (32). Indeed, detailed knowledge about the penetrance and age at onset of phenotypes associated with each disease, and the percentage of clinical cases accounted for by pathogenic variants in known genes are essential prerequisites for interpreting variants effectively (93). However, the majority of genetic diseases are so rare that it is difficult to compile specific “ground truth” datasets for tool assessment. This prompted us to investigate how the best tools for HCM perform in a disease-agnostic context (Figure 6C). Despite a clear reduction in performance metrics, ClinPred and MISTIC ranked among the five top tools being able to discriminate between pathogenic and benign missense variants with high sensitivity and specificity (>85%).

In conclusion, this study provides an objective framework for selecting the best-performing computational predictors to assist clinical interpretation of unknown missense variants. The results reported here may lay the foundation for a more consistent, reproducible and transparent approach to variant prediction across clinical diagnostic centers.

## Limitations of the study

The robustness of the analysis described in this study is highly dependent of the number and gene distribution of variants in the test datasets. In the pathogenic datasets used here, there is an over-representation of pathogenic variants in the *MYH7* gene because the vast majority of missense variation in HCM occurs in this gene. In contrast, variants in the benign datasets are more uniformly distributed throughout different genes. As the performance metrics combines the scores for both pathogenic and benign variants, the potential bias related to *MYH7* over-representation is in part counterbalanced. We did not evaluate prediction tools with high-throughput functional assays such as deep mutational scanning (30, 94) due to lack of HCM-specific data. Finally, our circularity-resilient analysis was limited to those tools for which the training datasets were available.

## Data availability statement

Publicly available datasets were analyzed in this study. This data can be found here: https://github.com/PedroBarbosa/paper_HCM_benchmark.

## Author contributions

MC-F, AF, and PB conceptualized and designed the study and wrote the manuscript. PB and MR collected and assembled the datasets. PB, MR, AF, and MC-F analyzed the data and interpreted the results. All authors contributed to the article and approved the submitted version.

## Funding

This work was supported by Fundação para a Ciência e a Tecnologia (FCT), Portugal (Fellowship SFRH/BD/137062/2018 to PB, and research support to LASIGE, UIDB/00408/2020), by FEDER/POR Lisboa 2020-Programa Operacional Regional de Lisboa, PORTUGAL 2020 (Infogene, 045300; CAMELOT, LISBOA-01-0247-FEDER-045915), and la Caixa Foundation under the agreement LCF/PR/HR20/52400021.

## Conflict of interest

Author MC-F is a cofounder and scientific advisor of GenoMed S.A., a molecular diagnosis company. Author AF is a consultant to GenoMed S.A. The remaining authors declare that the research was conducted in the absence of any commercial or financial relationships that could be construed as a potential conflict of interest.

## Publisher's note

All claims expressed in this article are solely those of the authors and do not necessarily represent those of their affiliated organizations, or those of the publisher, the editors and the reviewers. Any product that may be evaluated in this article, or claim that may be made by its manufacturer, is not guaranteed or endorsed by the publisher.

## Supplementary material

Supplementary Figure S1 Fraction of missing predictions per variant class in all variants evaluated (Clinvar HCM, SHaRe and Walsh_2017 datasets combined). Cardiac-specific methods are highlighted.

Supplementary Figure S2 Pearson correlation values between predictions of top-performant tools on all variants evaluated (Clinvar HCM, SHaRe and Walsh_2017 datasets combined).

Supplementary Table S1 Clinvar HCM-associated missense variants used in the study.

Supplementary Table S2 Variants used in the evaluation of SHaRe dataset. Pathogenic variants were selected from the SHaRe registry. Benign variants were selected from gnomAD v2.1.

Supplementary Table S3 Variants used in the evaluation of Walsh_2017 dataset. Pathogenic variants were selected from the Walch et al., 2017 paper. Benign variants were selected from gnomAD v2.1.

Supplementary Table S4 Variants obtained after tackling type 1 circularity issues of top-10 performing tools in Figure 5. Referred in the manuscript as “excluded_training_top_tools set”.

Supplementary Table S5 ClinVar variants reported after the development of all the top-10 performing tools in Figure 5. Referred in the manuscript as “ClinVar_recent set”.

Supplementary Table S6 Whole ClinVar missense variants after addressing type-1 circularity issues of all the top-10 performing tools in Figure 5. Dataset used to evaluate prediction tools irrespective of disease context (Figure 6C).

Supplementary Table S7 HCM-associated VUS used in the study.

## References

[1] Östman-Smith I Wettrell G Keeton B Holmgren D Ergander U Gould S. Age- and gender-specific mortality rates in childhood hypertrophic cardiomyopathy. Eur Heart J. (2008) 29:1160–7. 10.1093/eurheartj/ehn122 18385119

[2] Maron BJ Haas TS Ahluwalia A Murphy CJ Garberich RF. Demographics and epidemiology of sudden deaths in young competitive athletes: from the United States national registry. Am J Med. (2016) 129:1170–7. 10.1016/j.amjmed.2016.02.031 27039955

[3] Maron BJ Gardin JM Flack JM Gidding SS Kurosaki TT Bild DE. Prevalence of hypertrophic cardiomyopathy in a general population of young adults. Echocardiographic analysis of 4111 subjects in the CARDIA Study. Coronary Artery Risk Development in (Young) Adults. Circulation. (1995) 92:785–9. 10.1161/01.CIR.92.4.785 7641357

[4] Semsarian C Ingles J Maron MS Maron BJ. New perspectives on the prevalence of hypertrophic cardiomyopathy. J Am Coll Cardiol. (2015) 65:1249–54. 10.1016/j.jacc.2015.01.019 25814232

[5] Finocchiaro G Papadakis M Tanzarella G Dhutia H Miles C Tome M. Sudden death can be the first manifestation of hypertrophic cardiomyopathy: data from a United Kingdom pathology registry. JACC Clin Electrophysiol. (2019) 5:252–4. 10.1016/j.jacep.2018.11.004 30784699

[6] Kaski JP Norrish G Ding T Field E Ziółkowska L Olivotto I. Development of a novel risk prediction model for sudden cardiac death in childhood hypertrophic cardiomyopathy (HCM Risk-Kids). JAMA Cardiol. (2019) 4:918–27. 10.1093/eurheartj/ehz747.0062 31411652 PMC6694401

[7] Weissler-Snir A Allan K Cunningham K Connelly KA Lee DS Spears DA. Hypertrophic cardiomyopathy-related sudden cardiac death in young people in Ontario. Circulation. (2019) 140:1706–16. 10.1161/CIRCULATIONAHA.119.040271 31630535

[8] Hong Y Su WW Li X. Risk factors of sudden cardiac death in hypertrophic cardiomyopathy. Curr Opin Cardiol. (2022) 37:15–21. 10.1097/HCO.0000000000000939 34636345 PMC8654272

[9] Zamorano JL Anastasakis A Borger MA Borggrefe M Cecchi F Charron P. 2014 ESC Guidelines on diagnosis and management of hypertrophic cardiomyopathy: the Task Force for the Diagnosis and Management of Hypertrophic Cardiomyopathy of the European Society of Cardiology (ESC). Eur Heart J. (2014) 35:2733–79. 10.1093/eurheartj/ehu284 25173338

[10] Mazzarotto F Olivotto I Boschi B Girolami F Poggesi C Barton PJR. Contemporary insights into the genetics of hypertrophic cardiomyopathy: toward a new era in clinical testing? J Am Heart Assoc. (2020) 9:e015473. 10.1161/JAHA.119.015473 32306808 PMC7428545

[11] Hoss S Habib M Silver J Care M Chan RH Hanneman K. Genetic testing for diagnosis of hypertrophic cardiomyopathy mimics: yield and clinical significance. Circ Genomic Precis Med. (2020) 13:66–7. 10.1161/CIRCGEN.119.002748 32150461

[12] Charron P Arad M Arbustini E Basso C Bilinska Z Elliott P. Genetic counselling and testing in cardiomyopathies: a position statement of the European Society of Cardiology Working Group on Myocardial and Pericardial Diseases. Eur Heart J. (2010) 31:2715–28. 10.1093/eurheartj/ehq271 20823110

[13] Ackerman MJ Priori SG Willems S Berul C Brugada R Calkins H. HRS/EHRA expert consensus statement on the state of genetic testing for the channelopathies and cardiomyopathies this document was developed as a partnership between the Heart Rhythm Society (HRS) and the European Heart Rhythm Association (EHRA). Hear Rhythm. (2011) 8:1308–39. 10.1016/j.hrthm.2011.05.020 21787999

[14] Wolf CM. Hypertrophic cardiomyopathy: genetics and clinical perspectives. Cardiovasc Diagn Ther. (2019) 9(Suppl. 2):S388–415. 10.21037/cdt.2019.02.01 31737545 PMC6837941

[15] Christian S Cirino A Hansen B Harris S Murad AM Natoli JL. Diagnostic validity and clinical utility of genetic testing for hypertrophic cardiomyopathy: a systematic review and meta-analysis. Open Heart. (2022) 9:e001815. 10.1136/openhrt-2021-001815 35387861 PMC8987756

[16] Ingles J Burns C Bagnall RD Lam L Yeates L Sarina T. Nonfamilial hypertrophic cardiomyopathy: prevalence, natural history, and clinical implications. Circ Cardiovasc Genet. (2017) 10:e001620. 10.1161/CIRCGENETICS.116.001620 28408708

[17] Ho CY Day SM Ashley EA Michels M Pereira AC Jacoby D. Genotype and lifetime burden of disease in hypertrophic cardiomyopathy insights from the sarcomeric human cardiomyopathy registry (SHaRe). Circulation. (2018) 138:1387–98. 10.1161/CIRCULATIONAHA.117.033200 30297972 PMC6170149

[18] Richards S Aziz N Bale S Bick D Das S Gastier-Foster J. Standards and guidelines for the interpretation of sequence variants: a joint consensus recommendation of the American College of Medical Genetics and Genomics and the Association for Molecular Pathology. Genet Med. (2015) 17:405–24. 10.1038/gim.2015.30 25741868 PMC4544753

[19] Ghosh R Harrison SM Rehm HL Plon SE Biesecker LG. Updated recommendation for the benign stand-alone ACMG/AMP criterion. Hum Mutat. (2018) 39:1525–30. 10.1002/humu.23642 30311383 PMC6188666

[20] Harrison SM Biesecker LG Rehm HL. Overview of Specifications to the ACMG/AMP variant interpretation guidelines. Curr Protoc Hum Genet. (2019) 103:e93. 10.1002/cphg.93 31479589 PMC6885382

[21] Lek M Karczewski KJ Minikel E V Samocha KE Banks E Fennell T. Analysis of protein-coding genetic variation in 60,706 humans. Nature. (2016) 536:285–91. 10.1038/nature19057 27535533 PMC5018207

[22] Walsh R Thomson KL Ware JS Funke BH Woodley J McGuire KJ. Reassessment of Mendelian gene pathogenicity using 7,855 cardiomyopathy cases and 60,706 reference samples. Genet Med. (2017) 19:192–203. 10.1038/gim.2016.90 27532257 PMC5116235

[23] Thusberg J Olatubosun A Vihinen M. Performance of mutation pathogenicity prediction methods on missense variants. Hum Mutat. (2011) 32:358–68. 10.1002/humu.21445 21412949

[24] Walters-Sen LC Hashimoto S Thrush DL Reshmi S Gastier-Foster JM Astbury C. Variability in pathogenicity prediction programs: impact on clinical diagnostics. Mol Genet Genomic Med. (2015) 3:99–110. 10.1002/mgg3.116 25802880 PMC4367082

[25] Dong C Wei P Jian X Gibbs R Boerwinkle E Wang K. Comparison and integration of deleteriousness prediction methods for nonsynonymous SNVs in whole exome sequencing studies. Hum Mol Genet. (2015) 24:2125–37. 10.1093/hmg/ddu733 25552646 PMC4375422

[26] Mahmood K Jung C Philip G Georgeson P Chung J Pope BJ. Variant effect prediction tools assessed using independent, functional assay-based datasets: implications for discovery and diagnostics. Hum Genomics. (2017) 11:10. 10.1186/s40246-017-0104-8 28511696 PMC5433009

[27] Anderson D Lassmann T. A phenotype centric benchmark of variant prioritisation tools. npj Genomic Med. (2018) 3:5. 10.1038/s41525-018-0044-9 29423277 PMC5799157

[28] Li J Zhao T Zhang Y Zhang K Shi L Chen Y. Performance evaluation of pathogenicity-computation methods for missense variants. Nucleic Acids Res. (2018) 46:7793–804. 10.1093/nar/gky678 30060008 PMC6125674

[29] Hassan MS Shaalan AA Dessouky MI Abdelnaiem AE ElHefnawi M. Evaluation of computational techniques for predicting non-synonymous single nucleotide variants pathogenicity. Genomics. (2019) 111:869–82. 10.1016/j.ygeno.2018.05.013 29842949

[30] Livesey BJ Marsh JA. Using deep mutational scanning to benchmark variant effect predictors and identify disease mutations. Mol Syst Biol. (2020) 16:1–12. 10.15252/msb.20199380 32627955 PMC7336272

[31] Gunning AC Fryer V Fasham J Crosby AH Ellard S Baple EL. Assessing performance of pathogenicity predictors using clinically relevant variant datasets. J Med Genet. (2021) 58:547–55. 10.1136/jmedgenet-2020-107003 32843488 PMC8327323

[32] Zhang X Walsh R Whiffin N Buchan R Midwinter W Wilk A. Disease-specific variant pathogenicity prediction significantly improves variant interpretation in inherited cardiac conditions. Genet Med. (2021) 23:69–79. 10.1038/s41436-020-00972-3 33046849 PMC7790749

[33] Pejaver V Byrne AB Feng B-J Pagel KA Mooney SD Karchin R. Evidence-based calibration of computational tools for missense variant pathogenicity classification and ClinGen recommendations for clinical use of PP3/BP4 criteria. bioRxiv. (2022) 2022.03.17.484479. 10.1101/2022.03.17.484479 PMC9748256 36413997

[34] Liu X Li C Mou C Dong Y Tu Y. dbNSFP v4: a comprehensive database of transcript-specific functional predictions and annotations for human nonsynonymous and splice-site SNVs. Genome Med. (2020) 12:1–8. 10.1186/s13073-020-00803-9 33261662 PMC7709417

[35] Raney BJ Dreszer TR Barber GP Clawson H Fujita PA Wang T. Track data hubs enable visualization of user-defined genome-wide annotations on the UCSC Genome Browser. Bioinformatics. (2014) 30:1003–5. 10.1093/bioinformatics/btt637 24227676 PMC3967101

[36] McLaren W Gil L Hunt SE Riat HS Ritchie GRS Thormann A. The ensembl variant effect predictor. Genome Biol. (2016) 17:122. 10.1186/s13059-016-0974-4 27268795 PMC4893825

[37] Pedersen BS Layer RM Quinlan AR. Vcfanno: fast, flexible annotation of genetic variants. Genome Biol. (2016) 17:118. 10.1186/s13059-016-0973-5 27250555 PMC4888505

[38] Landrum MJ Lee JM Benson M Brown GR Chao C Chitipiralla S. ClinVar: improving access to variant interpretations and supporting evidence. Nucleic Acids Res. (2018) 46:D1062–7. 10.1093/nar/gkx1153 29165669 PMC5753237

[39] Louden DN. MedGen: NCBI's Portal to Information on Medical Conditions with a Genetic Component. Med Ref Serv Q. (2020) 39:183–91. 10.1080/02763869.2020.1726152 32329672

[40] Amberger JS Bocchini CA Schiettecatte F Scott AF Hamosh A. OMIMorg: Online Mendelian Inheritance in Man (OMIM^®^), an online catalog of human genes and genetic disorders. Nucleic Acids Res. (2015) 43:D789–98. 10.1093/nar/gku1205 25428349 PMC4383985

[41] Vasilevsky NA Matentzoglu NA Toro S Flack JE Hegde H Unni DR. Mondo: Unifying diseases for the world, by the world. medRxiv. (2022) 2022.04.13.22273750. 10.1101/2022.04.13.22273750

[42] Karczewski KJ Francioli LC Tiao G Cummings BB Alföldi J Wang Q. The mutational constraint spectrum quantified from variation in 141,456 humans. Nature. (2020) 581:434–43. 10.1530/ey.17.14.3 32461654 PMC7334197

[43] Pedersen BS Quinlan AR. cyvcf2: fast, flexible variant analysis with Python. Hancock J, editor Bioinformatics. (2017) 33:1867–9. 10.1093/bioinformatics/btx057 28165109 PMC5870853

[44] Wang M Callenberg KM Dalgleish R Fedtsov A Fox NK Freeman PJ. hgvs: A Python package for manipulating sequence variants using HGVS nomenclature: 2018 Update. Hum Mutat. (2018) 39:1803–13. 10.1002/humu.23615 30129167 PMC6282708

[45] Pedregosa F Varoquaux G Gramfort A Michel V Thirion B Grisel O. Scikit-learn: machine learning in python. J Mach Learn Res. (2012) 12:2825–30. 10.48550/arXiv.1201.0490

[46] Waskom M. Seaborn: statistical data visualization. J Open Source Softw. (2021) 6:3021. 10.21105/joss.03021

[47] Akhtar M Elliott P. The genetics of hypertrophic cardiomyopathy. Glob Cardiol Sci Pract. (2018) 2018:36. 10.21542/gcsp.2018.36 30393648 PMC6209452

[48] Kumar P Henikoff S Ng PC. Predicting the effects of coding non-synonymous variants on protein function using the SIFT algorithm. Nat Protoc. (2009) 4:1073–82. 10.1038/nprot.2009.86 19561590

[49] Li B Krishnan VG Mort ME Xin F Kamati KK Cooper DN. Automated inference of molecular mechanisms of disease from amino acid substitutions. Bioinformatics. (2009) 25:2744–50. 10.1093/bioinformatics/btp528 19734154 PMC3140805

[50] Adzhubei IA Schmidt S Peshkin L Ramensky VE Gerasimova A Bork P. A method and server for predicting damaging missense mutations. Nat Methods. (2010) 7:248–9. 10.1038/nmeth0410-248 20354512 PMC2855889

[51] Reva B Antipin Y Sander C. Predicting the functional impact of protein mutations: application to cancer genomics. Nucleic Acids Res. (2011) 39:e118. 10.1093/nar/gkr407 21727090 PMC3177186

[52] Liu X Wu C Li C Boerwinkle E. dbNSFP v3.0: a one-stop database of functional predictions and annotations for human non-synonymous and splice site SNVs. Hum Mutat. (2016) 37:235–41. 10.1002/humu.22932 26555599 PMC4752381

[53] González-Pérez A López-Bigas N. Improving the assessment of the outcome of nonsynonymous SNVs with a consensus deleteriousness score, Condel. Am J Hum Genet. (2011) 88:440–9. 10.1016/j.ajhg.2011.03.004 21457909 PMC3071923

[54] Carter H Douville C Stenson PD Cooper DN Karchin R. Identifying Mendelian disease genes with the variant effect scoring tool. BMC Genomics. (2013) 14 (Suppl. 3):S3. 10.1186/1471-2164-14-S3-S3 23819870 PMC3665549

[55] Schwarz JM Cooper DN Schuelke M Seelow D. Mutationtaster2: mutation prediction for the deep-sequencing age. Nat Methods. (2014) 11:361–2. 10.1038/nmeth.2890 24681721

[56] Shihab HA Gough J Cooper DN Stenson PD Barker GLA Edwards KJ. Predicting the functional, molecular, and phenotypic consequences of amino acid substitutions using hidden markov models. Hum Mutat. (2013) 34:57–65. 10.1002/humu.22225 23033316 PMC3558800

[57] Choi Y Chan AP. PROVEAN web server: a tool to predict the functional effect of amino acid substitutions and indels. Bioinformatics. (2015) 31:2745–7. 10.1093/bioinformatics/btv195 25851949 PMC4528627

[58] Jagadeesh KA Wenger AM Berger MJ Guturu H Stenson PD Cooper DN. M-CAP eliminates a majority of variants of uncertain significance in clinical exomes at high sensitivity. Nat Genet. (2016) 48:1581–6. 10.1038/ng.3703 27776117

[59] Ioannidis NM Rothstein JH Pejaver V Middha S McDonnell SK Baheti S. REVEL: an ensemble method for predicting the pathogenicity of rare missense variants. Am J Hum Genet. (2016) 99:877. 10.1016/j.ajhg.2016.08.016 27666373 PMC5065685

[60] Samocha KE Kosmicki JA Karczewski KJ O'Donnell-Luria AH Pierce-Hoffman E MacArthur DG. Regional missense constraint improves variant deleteriousness prediction. bioRxivM. (2017) 12:148353. 10.1101/148353

[61] Traynelis J Silk M Wang Q Berkovic SF Liu L Ascher DB. Optimizing genomic medicine in epilepsy through a gene-customized approach to missense variant interpretation. Genome Res. (2017) 27:1715–29. 10.1101/gr.226589.117 28864458 PMC5630035

[62] Sundaram L Gao H Padigepati SR McRae JF Li Y Kosmicki JA. Predicting the clinical impact of human mutation with deep neural networks. Nat Genet. (2018) 50:1161–70. 10.1038/s41588-018-0167-z 30038395 PMC6237276

[63] Alirezaie N Kernohan KD Hartley T Majewski J Hocking TD. ClinPred: prediction tool to identify disease-relevant nonsynonymous single-nucleotide variants. Am J Hum Genet. (2018) 103:474–83. 10.1016/j.ajhg.2018.08.005 30220433 PMC6174354

[64] Chennen K Weber T Lornage X Kress A Böhm J Thompson J. MISTIC: A prediction tool to reveal disease-relevant deleterious missense variants. PLoS ONE. (2020) 15:e0236962. 10.1371/journal.pone.0236962 32735577 PMC7394404

[65] Jaravine V Balmford J Metzger P Boerries M Binder H Böker M. Annotation of human exome gene variants with consensus pathogenicity. Genes. (2020) 11:1–18. 10.3390/genes11091076 32938008 PMC7563776

[66] Qi H Zhang H Zhao Y Chen C Long JJ Chung WK. MVP predicts the pathogenicity of missense variants by deep learning. Nat Commun. (2021) 12:1–9. 10.1038/s41467-020-20847-0 33479230 PMC7820281

[67] Wu Y Li R Sun S Weile J Roth FP. Improved pathogenicity prediction for rare human missense variants. Am J Hum Genet. (2021) 108:1891–906. 10.1016/j.ajhg.2021.08.012 34551312 PMC8546039

[68] Jiang T Wang K Fang L. MutFormer: A context-dependent transformer-based model to predict pathogenic missense mutations. arXiv [Preprint]. (2021). 10.48550/ARXIV.2110.14746

[69] Frazer J Notin P Dias M Gomez A Min JK Brock K. Disease variant prediction with deep generative models of evolutionary data. Nature. (2021) 599:91–5. 10.1038/s41586-021-04043-8 34707284

[70] Quinodoz M Peter VG Cisarova K Royer-Bertrand B Stenson PD Cooper DN. Analysis of missense variants in the human genome reveals widespread gene-specific clustering and improves prediction of pathogenicity. Am J Hum Genet. (2022) 109:457. 10.1016/j.ajhg.2022.01.006 35120630 PMC8948164

[71] Siepel A. Evolutionarily conserved elements in vertebrate, insect, worm, and yeast genomes. Genome Res. (2005) 15:1034–50. 10.1101/gr.3715005 16024819 PMC1182216

[72] Siepel A Pollard KS Haussler D. New methods for detecting lineage-specific selection. In: Research in Computational Molecular Biology. Berlin; Heidelberg: Springer (2006). p. 190–205.

[73] Garber M Guttman M Clamp M Zody MC Friedman N Xie X. Identifying novel constrained elements by exploiting biased substitution patterns. Bioinformatics. (2009) 25:i54–62. 10.1093/bioinformatics/btp190 19478016 PMC2687944

[74] Davydov E V Goode DL Sirota M Cooper GM Sidow A Batzoglou S. Identifying a High Fraction of the Human Genome to be under Selective Constraint Using GERP++. PLoS Comput Biol. (2010) 6:e1001025. 10.1371/journal.pcbi.1001025 21152010 PMC2996323

[75] Di Iulio J Bartha I Wong EHMM Yu H-CC Lavrenko V Yang D. The human noncoding genome defined by genetic diversity. Nat Genet. (2018) 50:333–7. 10.1038/s41588-018-0062-7 29483654

[76] Ritchie GRS Dunham I Zeggini E Flicek P. Functional annotation of noncoding sequence variants. Nat Methods. (2014) 11:294–6. 10.1038/nmeth.2832 24487584 PMC5015703

[77] Bendl J Musil M Štourač J Zendulka J Damborský J Brezovský J. PredictSNP2: a unified platform for accurately evaluating SNP effects by exploiting the different characteristics of variants in distinct genomic regions. PLOS Comput Biol. (2016) 12:e1004962. 10.1371/journal.pcbi.1004962 27224906 PMC4880439

[78] Shihab HA Rogers MF Gough J Mort M Cooper DN Day INM. An integrative approach to predicting the functional effects of non-coding and coding sequence variation. Bioinformatics. (2015) 31:1536–43. 10.1093/bioinformatics/btv009 25583119 PMC4426838

[79] Quang D Chen Y Xie X. DANN: a deep learning approach for annotating the pathogenicity of genetic variants. Bioinformatics. (2015) 31:761–3. 10.1093/bioinformatics/btu703 25338716 PMC4341060

[80] Richardson TG Campbell C Timpson NJ Gaunt TR. Incorporating non-coding annotations into rare variant analysis. PLoS ONE. (2016) 11:e0154181. 10.1371/journal.pone.0154181 27128317 PMC4851421

[81] Ionita-Laza I McCallum K Xu B Buxbaum JD. A spectral approach integrating functional genomic annotations for coding and noncoding variants. Nat Genet. (2016) 48:214–20. 10.1038/ng.3477 26727659 PMC4731313

[82] Smedley D Schubach M Jacobsen JOB Köhler S Zemojtel T Spielmann M. A whole-genome analysis framework for effective identification of pathogenic regulatory variants in mendelian disease. Am J Hum Genet. (2016) 99:595–606. 10.1016/j.ajhg.2016.07.005 27569544 PMC5011059

[83] Schubach M. ReMM Threshold. (2018) Available online at: https://github.com/exomiser/Exomiser/issues/268 (accessed May 28, 2018).

[84] Li S Van Der Velde KJ De Ridder D Van Dijk ADJ Soudis D Zwerwer LR. CAPICE: a computational method for consequence-agnostic pathogenicity interpretation of clinical exome variations. Genome Med. (2020) 12:1–11. 10.1186/s13073-020-00775-w 32831124 PMC7446154

[85] Rentzsch P Schubach M Shendure J Kircher M. CADD-Splice—improving genome-wide variant effect prediction using deep learning-derived splice scores. Genome Med. (2021) 13:1–12. 10.1186/s13073-021-00835-9 33618777 PMC7901104

[86] Nicora G Limongelli I Gambelli P Memmi M Malovini A Mazzanti A. CardioVAI: An automatic implementation of ACMG-AMP variant interpretation guidelines in the diagnosis of cardiovascular diseases. Hum Mutat. (2018) 39:1835–46. 10.1002/humu.23665 30298955

[87] Kelly MA Caleshu C Morales A Buchan J Wolf Z Harrison SM. Adaptation and validation of the ACMG/AMP variant classification framework for MYH7-associated inherited cardiomyopathies: recommendations by ClinGen's Inherited Cardiomyopathy Expert Panel. Genet Med. (2018) 20:351–9. 10.1038/gim.2017.218 29300372 PMC5876064

[88] Grimm DG Azencott C-A Aicheler F Gieraths U MacArthur DG Samocha KE. The evaluation of tools used to predict the impact of missense variants is hindered by two types of circularity. Hum Mutat. (2015) 36:513–23. 10.1002/humu.22768 25684150 PMC4409520

[89] Stenson PD Mort M Ball E V Chapman M Evans K Azevedo L. The Human Gene Mutation Database (HGMD^®^): optimizing its use in a clinical diagnostic or research setting. Hum Genet. (2020) 139:1197. 10.1007/s00439-020-02199-3 32596782 PMC7497289

[90] Tian Y Pesaran T Chamberlin A Fenwick RB Li S Gau CL. REVEL and BayesDel outperform other in silico meta-predictors for clinical variant classification. Sci Reports. (2019) 9:1–6. 10.1038/s41598-019-49224-8 31484976 PMC6726608

[91] Pollard S Sun S Regier DA. Balancing uncertainty with patient autonomy in precision medicine. Nat Rev Genet. (2019) 20:251–2. 10.1038/s41576-019-0111-9 30872766

[92] Tsai GJ Rañola JMO Smith C Garrett LT Bergquist T Casadei S. Outcomes of 92 patient-driven family studies for reclassification of variants of uncertain significance. Genet Med. (2019) 21:1435–42. 10.1038/s41436-018-0335-7 30374176

[93] Nykamp K Anderson M Powers M Garcia J Herrera B Ho YY. Sherloc: a comprehensive refinement of the ACMG-AMP variant classification criteria. Genet Med. (2017) 19:1105–17. 10.1038/gim.2017.37 28492532 PMC5632818

[94] Cubuk C Garrett A Choi S King L Loveday C Torr B. Clinical likelihood ratios and balanced accuracy for 44 in silico tools against multiple large-scale functional assays of cancer susceptibility genes. Genet Med. (2021) 23:2096–104. 10.1038/s41436-021-01265-z 34230640 PMC8553612

